# Dioxin and dibenzofuran like molecular analogues from the pyrolysis of biomass materials—the emerging challenge in bio-oil production

**DOI:** 10.1186/s13065-020-00732-z

**Published:** 2021-01-15

**Authors:** Samuel K. Kirkok, Joshua K. Kibet, Thomas Kinyanjui, Francis I. Okanga, Vincent O. Nyamori

**Affiliations:** 1 Department of Chemistry, Egerton University, P.O Box 536, Egerton, 20115 Kenya; 2 School of Chemistry and Physics, University of KwaZulu-Natal, Westville Campus, Private Bag X54001, Durban, 4000 South Africa

**Keywords:** Bio-oil, Dibenzofuran-like analogues, Cross-reactions, Emerging pollutants, Free radical species

## Abstract

**Introduction:**

The aggressive search for renewable energy resources and essential pyrosynthetic compounds has marked an exponential rise in the thermal degradation of biomass materials. Consequently, clean and sustainable transport fuels are increasingly desirable in a highly industrialized economy, for energy security and environmental protection. For this reason, biomass materials have been identified as promising alternatives to fossil fuels despite the challenges resulting from the possible formation of toxic nitrogen-based molecules during biomass degradation. In order to understand the free radical characteristic challenges facing the use of bio-oil, a brief review of the effects of free radicals in bio-oil is presented.

**Methodology:**

Pyrolysis was conducted in a tubular flow quartz reactor at a residence time of 2 s at 1 atm. pressure, for a total pyrolysis time of 5 min. The thermal degradation of biomass components was investigated over the temperature range of 200 to 700 °C typically in 50 °C increments under two reaction conditions; pyrolysis in N_2_ and oxidative pyrolysis in 5% O_2_ in N_2_. The pyrolysate effluent was analysed using a Gas chromatograph hyphenated to a mass selective detector (MSD).

**Results:**

The yield of levoglucosan in the pyrolysis of cellulose in the entire pyrolysis temperature range was 68.2 wt % under inert conditions and 28.8 wt % under oxidative conditions. On the other hand, formaldehyde from pyrolysis of cellulose yielded 4 wt % while that from oxidative pyrolysis was 7 wt % translating to ⁓ 1.8 times higher than the yield from pyrolysis. Accordingly, we present for the first time dioxin-like and dibenzofuran-like nitrogenated analogues from an equimassic pyrolysis of cellulose and tyrosine. Levoglucosan and formaldehyde were completely inhibited during the equimassic pyrolysis of cellulose and tyrosine.

**Conclusion:**

Clearly, any small amounts of N-biomass components such as amino acids in cellulosic biomass materials can inhibit the formation of levoglucosan–a major constituent of bio-oil. Overall, a judicious balance between the production of bio-oil and side products resulting from amino acids present in plant matter should be taken into account to minimize economic losses and mitigate against negative public health concerns.
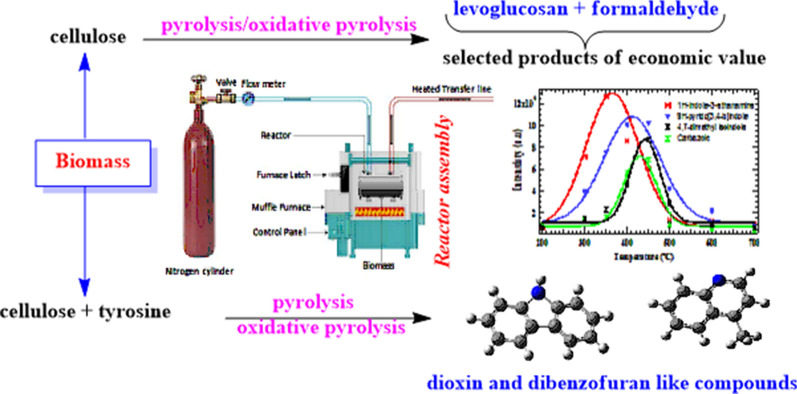

## Introduction

The International Agency for Research on Cancer (IARC) has stressed that, on long-term exposure, emissions from fossil operated engines are carcinogenic to both humans and animals [1]. The main motivation in this paper is to conduct the pyrolysis of biomass materials, mainly cellulose, amino acids and binary mixtures of cellulose and amino acids with a view to understanding the thermal degradation characteristics of these biomass resources and their health, environmental, and technological impacts especially in the production of bio-oil (Cervantes et al. 2020; [2]). The transformational processes of model biomass components to bio-oil and other reaction products of technological importance are of great interest in providing insight into cross-reactions that occur during the thermal degradation of biomass materials. Therefore, sustainable development with minimum environmental degradation and better economic capacity has been the primary emphasis in many countries with growing attention in the development of technologies aimed at harnessing renewable energy resources [3].

The thermal processing of biomass materials arguably represents a promising technique for the recovery of renewable energy resources at the industrial level, but the complicated structural composition of biomass makes it difficult to link the formation of organic pollutants to specific chemical reactions without the use of model or surrogate biomass compounds [4]. Model compounds provide a clear understanding of the reaction processes and potential reaction products evolved during the thermal degradation of biomass components either for economic purposes or for environmental management. Lately, of interest to the combustion community is the interaction of N-biomass with other biomass components such as cellulose. Ideally, nitrogen in biomass is mainly in the form of proteins and amino acids. Also, it is widely accepted that the combined study of biomass and nitrogen-containing model compounds, such as amino acids and protein, is probably the best approach to gain insight into a comprehensive mechanism of N-biomass chemistry [5, 6]. N-biomass is of significant importance because of the emissions of poisonous gases, such as hydrogen cyanide, NOx and cyanogen (HNCO), which may outweigh the benefits of biofuel production and the synthesis of essential chemicals through thermochemical processes (Ren and Zhao [7]).

### Environmentally persistent free radicals (EPFRs) in bio-oil

Electron paramagnetic resonance (EPR) spectroscopy is an analytical method to explore materials with un-paired electrons, and, therefore it is an important technique to probe the presence of radicals in the bio-oil matrix [8, 9]. Conventionally, EPR measures energy absorption attributed to the transition of subatomic particles such as electrons between different energy levels caused by the interaction of free radicals with the magnetic field component of the microwave radiation in presence of an external magnetic field applied to the sample [9, 10]. EPR has been used broadly to study free radicals in various condensed phases such as biochar [11, 8].

**Scheme 1:**
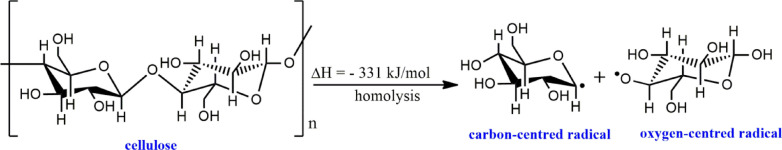
Proposed free radical formation from the thermal degradation of cellulose– modified from [Zhang et al. [13]]

Lately, studies have indicated the presence of reactive free radical species in bio-oil proposed to degrade the quality of bio-oil [3]. Therefore, bio-oil run in vehicular systems or internal combustion engines can trigger the emission of enormous free radical species, particularly in the form of thermal particulates, according to Mosonik et al. [12]. Previous studies have also shown that bio-oils produced from lignocellulosic materials exhibit evidence of homolytic bond cleavage (Scheme 1) during pyrolysis, which results in the generation of free radicals in the order of 7.5 × 10^20^ spins g^−1^ – 5.4 × 10^18^ spins g^−1^, and this could be mainly carbon-centred (benzyl) or oxygen-centred (phenoxy) radical species delocalized within a highly conjugated π–π system, implying that they are very stable [9, [8].

Table 1 presents the free radical characteristics of various bio-oil and biodiesel reported in the literature. The bond dissociation energy (BDE) for homolysis of cellulose was evaluated by Zhang et al. [13] at absolute 0 K using the density functional theory (DFT) formalism before and after cleavage and found that the C—O BDE for homolytic cleavage was 331 kJ mol^−1^. This process suggests the yields of two types of radical species (cf. Scheme 1), i.e., carbon-centred and oxygen–centred radicals, as being present in cellulose bio-oil. Consequently, levoglucosan, which is largely the main product of cellulose pyrolysis, is unlikely to form free radical species because of its high energy barrier, according to Zhang et al. [13].

**Table 1:** The free radical characteristics of bio-oil and biodiesel

Bio-oil/biodiesel	$\Delta {\mathbf{H}}_{{{\mathbf{P}} - {\mathbf{P}}}}$	${\mathbf{g}} - {\mathbf{factor}}$	Nature of radical	Ref.
Loblolly pine bio-oil	3.2 – 5.2	2.0026 – 2.0033	Carbon/phenoxy–centered	[8]
Cellulose bio-oil	5.01	2.0031	Carbon/oxygen –centered	[9]
Lignin bio-oil	4.92	2.0030	Carbon/phenoxy–centered	[9]
Corn-Stover bio-oil	5.48	2.0028	Carbon-centered	[9]
Corn-Stover bio-oil PL	2.35	2.0028	Carbon-centered	[9]
Commercial biodiesel 100	5.2	2.006	Oxygen-centered	[3]
Commercial biodiesel	0.61	2.007	Oxygen-centered	[41]

*PL* pyrolytic lignin

The production of levoglucosan from the thermal degradation of cellulose, a major constituent of bio-oil, is given detailed treatment in this study. Moreover, formaldehyde is discussed briefly as an important precursor for the manufacture of drugs, vaccines, and resins, notwithstanding its carcinogenic potential when exposed to humans. It is important to note that other major products of cellulose pyrolysis, including furan, furfural, 5-hydroxymethyl furfural, hydroxyacetaldehyde, and hydroxyacetone, have received significant attention in the literature and, therefore, will not be the subject of further treatment in this work [14, 15]. In addition, because thermal char is an important product of cellulose pyrolysis, its formation characteristics will be described in the light of soil amendment and carbon sequestration. The challenges facing bio-oil production owing to the formation of legacy pollutants such as dioxin and dibenzofuran-like nitrogenated analogues are also discussed in this work. The yield of formaldehyde from the thermal degradation of biomass is scarce in the literature despite its critical role in industrial processes; pharmaceuticals, and resins, among other applications. Even though levoglucosan is presently overlooked because of its high cost during synthesis and purification, it has a significant potential for commercial applications such as the synthesis of bio-oil, polymers, solvents and medicinal intermediates for the manufacture of drugs [16]. Few studies exist in literature that elucidate on the cross-reaction processes occurring during the co-pyrolysis of cellulosic materials and amino acids aimed at understanding the effect of N-chemistry in bio-oil production, its applications, and the factors precipitating its unsuitability for use as a transport fuel.

## Materials and methods

### Materials and experimental procedure

The biomass samples; tyrosine, and cellulose (purity ˃99%) explored in this study were procured from Sigma Aldrich (USA) and used without additional treatment. In order to avoid many experimental drawbacks associated with the analysis of biomass pyrolysis, the system for thermal diagnostic studies (STDS) was used. The system for thermal diagnostic studies (STDS) is a continuous flow reactor system using an in-line GCMS at the head of the GC column at − 60 °C. It is a well-tested technique for the analysis of a broad range of organic materials, including amino acids Nganai et al. [17]. The experimental details for STDS are reported elsewhere [18]. The thermal degradation of biomass components was generally investigated in a tubular flow reactor over the temperature range of 200 to 700 °C at 1 atmospheric pressure, typically in 50 °C increments under two reaction conditions; pyrolysis in N_2_ and oxidative pyrolysis in 5% O_2_ in N_2_. The concentration of oxygen in the oxidative pyrolysis experiments was kept low to optimize the formation of molecular products. Generally, fractional pyrolysis is applied as an experimental technique in which the same sample is continuously pyrolyzed at each pyrolysis temperature [19]. This is a selective in situ conversion of biopolymers to molecular products [6]. In this work, the gas flow rate was designed to maintain a constant residence time of 2 s. The sample, 50 ± 0.2 mg, was loaded into the tubular quartz reactor of dimensions 0.3 cm i.d. x 17.7 cm length which was held in place by quartz wool. The reactor containing the sample was then placed inside an electrically heated furnace for a total pyrolysis time of 5 min while the heating rate was kept isothermally at 10 °C s^−1^. At the end of 5 min pyrolysis time, the furnace was turned off and the sample exposed to flowing N_2_ and the cooling furnace turned on to cool the sample system. The reactor used was fabricated in our laboratory. Experimental data presented were averaged values from 3 replicates. The reactor assembly applied in this study is presented in the Additional file 1 section, Figure S1.

### Characterization of molecular products

The gas chromatograph-mass spectrometry (GCMS) analysis of pyrolysate effluent was performed using an Agilent 6890 N gas chromatograph equipped with a 5973 N mass selective detector (MSD) with an electron impact (EI) ion source of 70 eV. A DB5-MS GC column (30 m x 0.25 mm x 0.25 µm) was used in this study. The temperature program was typical, i.e., 60 °C initial temperature; held for 3 min followed by a heating rate of 15 °C min^−1^ to 150 °C; held for 1 min, and the temperature raised to 300 °C at a rate of 5 °C min^−1^ and held for 5 min. The injector and MSD detector temperatures were kept at 250 °C and 280 °C, respectively. Ultra-high pure (UHP, 99.999%) helium was used as the carrier gas at a constant flow of 3.3 mL min^−1^. The mass spectrometer was operated in total ion current mode (TIC) over a mass scan range of 26 to 500 atomic mass units (amu). The analytes were run through NIST and ChemStation softwares for identification. To guarantee the validity of the data, pure compounds were run through the GCMS system under similar conditions as the sample and recoveries of between 99 and 106% were obtained. These recoveries were sufficient to qualify the analytical data reported in this study [20]. Moreover, calibration of molecular products and the weight percent yield calculations; structural formulas for the products from the biomass matrix (cellulose and tyrosine) pyrolysis/oxidative pyrolysis are reported in Additional file 1: Figures S2, S3 and S4.

## Results and discussion

**Fig. 1:**
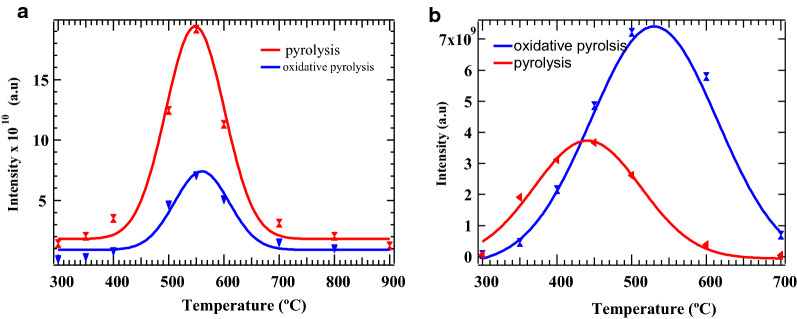
Yields of **a** levoglucosan and **b** formaldehyde (wt %) from pyrolysis and oxidative pyrolysis of cellulose, respectively

Herein we focus on levoglucosan and formaldehyde as key products from the thermal degradation of cellulose, which have attracted mounting research interest because of their important industrial applications [21, 13]. Herein, Fig. 1, we present the product yields of levoglucosan and formaldehyde as a function of temperature from the thermal degradation of cellulose under an inert environment (N_2_) and in an oxidative atmosphere (5% O_2_ in N_2_) as conducted in our laboratory under conditions that support thermochemical conversion. As can be observed in Fig. 1a, it is evident that under purely inert conditions, the yield of levoglucosan from pyrolysis of cellulose in the entire temperature range was ⁓ 2.5 times higher than that from oxidative pyrolysis. The yield of levoglucosan derived from cellulose pyrolysis in this work was 68.2 wt %, whereas the yield obtained from oxidative pyrolysis was 28.8 wt %. On the other hand, formaldehyde from pyrolysis of cellulose yielded 4% wt % while that from oxidative pyrolysis was 7 wt % translating to ⁓ 1.8 times higher than the yield from pyrolysis, Fig. 1b.

The difference in the yields of levoglucosan from the two pyrolysis conditions can be explained in terms of the reactivity of the •H and the •OH radicals present in the pyrolysis pool. Hydroxyl radical (•OH) is suggested to play an important role during the oxidative pyrolysis of cellulose and thus influences the reaction products observed in Fig. 1. The rate of formation of formaldehyde is enhanced for oxidative pyrolysis because the process takes place under a reactive environment, in the presence of O_2_ and •OH and this explains why the concentration of formaldehyde for oxidative pyrolysis experiment is much higher than that of pyrolysis experiment [18]. Also, it is expected that •OH, as the main chain carrier, would enhance the formation of levoglucosan, but this is not the case since its formation basically involves the unzipping of the cellulose chains followed by homolytic bond cleavage [13]. Moreover, levoglucosan is proposed to form via intramolecular mechanism and condensation reactions followed by a series of depolymerization of the glycosidic units [22]. Vinu et al. [23] and Bai et al. [22] proposed that the primary thermal deconstruction of cellulose to levoglucosan occurs predominantly via a concerted mechanistic process [24]. Nevertheless, the mechanistic formation of levoglucosan from cellulose will not be discussed in this study as it has already been treated in detail elsewhere [13, 22]. Summarily, an oxidative atmosphere favours the formation of formaldehyde, whereas a pyrolytic atmosphere enhances the formation of levoglucosan. This implies that, for industrial benefits, a purely inert atmosphere should be employed in the production of levoglucosan, anhydro-β-d-glucopyranose,– a major component of bio-oil and other essential chemicals, and evidently the primary product from the thermal decomposition of cellulose [16, 25, 26]. The molecular structure of levoglucosan is presented in the Additional file 1: Figure S2.

### Thermal char from the thermal degradation of cellulose relative to the formation of levoglucosan

Among other conventional industrial uses such as purification of water; the major use of biochar is soil amendment, thus slowly replacing artificial fertilizers which are not environmentally friendly [27]. For this reason therefore, the production of biochar from biomass degradation is not only of economic significance but also plays an important role in carbon sequestration to maintain carbon balance and mitigate against global warming and environmental degradation [28]. Accordingly, the decomposition of cellulose occurs slowly at 200 °C and reaches a maximum at 400 °C, according to a study performed in our laboratory. There is an abrupt loss in mass from 300 to 400 °C, with approximately 60% loss in mass. This is a clear indication that cellulose decomposes easily with an increase in temperature, yielding predominantly levoglucosan (a major component of bio-oil)–Fig. 1a, b.

**Fig. 2:**
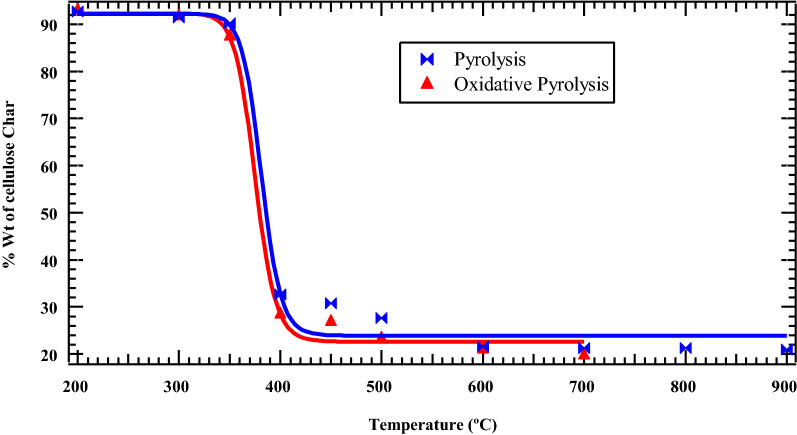
Weight % cellulose as a function of temperature

Char yields decreased significantly as the pyrolysis temperature is increased. Notably, levoglucosan boils at 400 °C [29], and its evaporation contributes to the sharp mass loss during cellulose pyrolysis (cf. Fig. 2). Between 400 °C and 600 °C, where the concentration of levoglucosan is highest (cf. Fig. 1), the mass loss in cellulose for both pyrolysis and oxidation experiments is over 70%. With reference to Fig. 2, there was no major difference in mass loss between pyrolysis and oxidative pyrolysis of cellulose. For instance, at 200 °C, the mass loss for pyrolysis and oxidative pyrolysis was 8.3 and 8.1%, respectively, while at 400 °C, the mass loss was 78.7 and 80.3%, respectively. Two decomposition regimes are observed for cellulose: the first decomposition regime occurs between 200 and 400 °C while the second decomposition regime occurs above 400 °C (between 400 °C and 900 °C for pyrolysis, and between 400 °C and 700 °C for oxidative pyrolysis).

It is evident from Fig. 2 that cellulose does not yield a high amount of thermal char as compared to other biomass materials such as hemicellulose and lignin as reported elsewhere [10, 30]. Therefore, it is a better biomass material to explore for the commercial production of bio-oil and other essential chemicals for use in polymer synthesis and pharmaceuticals. Particularly, char is formed at low temperature and can undergo a chemical transformation at a temperature greater than 400 °C to form a more stable and relatively ordered carbon structure, although it may contain detrimental environmentally persistent free radicals [12, 31].

### The impacts of dioxin-dibenzo furan like compounds in the pyrosynthesis of bio-oil

As the concerns for energy supply and pollution problems caused by burning fossil fuels become more disturbing, rising attention has been focused towards the use of renewable and clean energy combustion of biomass materials, but the shift in this direction is not without challenges motivated by serious environmental pollutants including mutagenic nitrogenated heterocycles [6, 32]. The major concern in the pyrolysis of amino acids in the presence of cellulosic materials is the formation of mutagenic compounds such as heterocyclic amines formed at elevated temperatures [6, 33]. Nitrogen transformation during the co-pyrolysis of amino acid with other biomass components at different ratios determines the interaction characteristics of cellulosic material with N-biomass chemistry (Ren et al. [34, 35]). The result of their study on the co-pyrolysis of amino acids and lignocellulose underlines the emissions of hazardous gas-phase products such as hydrogen cyanide (HCN) and cyanogen (HNCO). Unfortunately, this is not beneficial for clean energy combustion technologies.

**Fig. 3:**
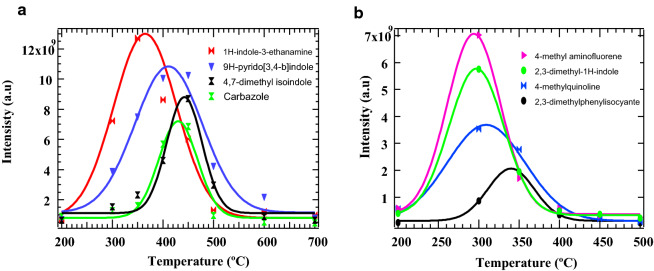
Major products from the co-pyrolysis (**a**) and **b** co-oxidative pyrolysis of cellulose and tyrosine

Nonetheless, one fundamental gap in the experiments performed by Ren and his co-workers (2011; 2013) was the absence of an in-depth determination and discussion of high molecular weight pyrolysis products of environmental concern. The reason given for this observation is that the formation of high molecular weight products from the pyrolysis of small amino acid compounds is not common in literature due to their decomposition nature, which implies an elimination reaction according to Cervantes et al. (2020). Therefore, this work provides experimental data that will shed more light on the formation of high molecular weight compounds from the co-pyrolysis of tyrosine and cellulose. From Fig. 3a, it is evident that the nitrogenated molecular compounds reported are dibenzofuran-like chemicals and their associated dioxin compounds. For many decades, these compounds have been considered as extremely toxic especially when halogenated in the presence of a transition metal catalyst such as iron or copper [10, 36]. From a toxicological perspective, these dioxin-like and dibenzofuran-like analogues are considered emerging environmental pollutants and are a burden to public health systems [37].

**Fig. 4:**
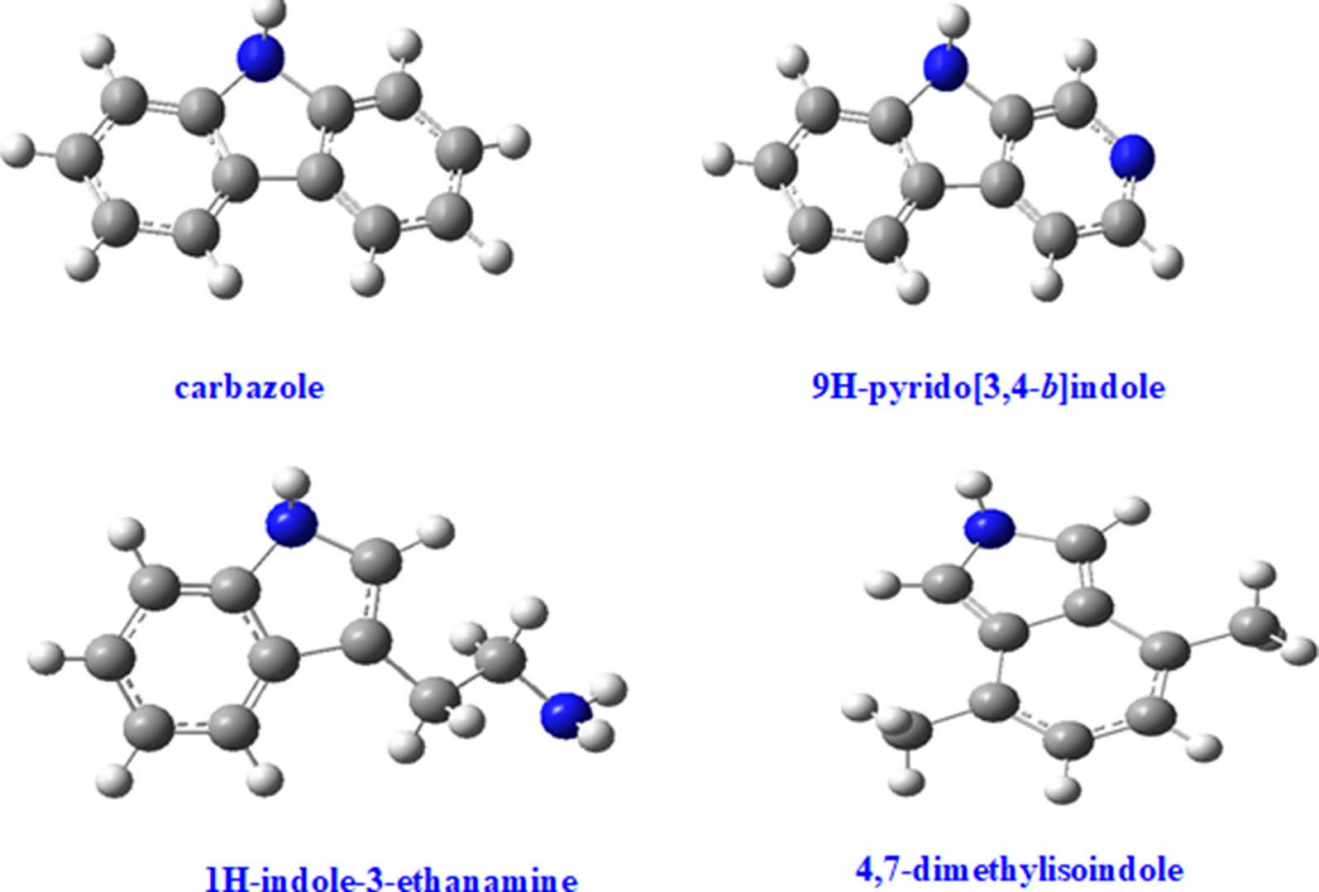
Reaction products from the pyrolysis of equimassic mixture of cellulose and tyrosine-the blue atoms represent nitrogen; grey atoms are carbons while white atoms are hydrogens

**Fig. 5:**
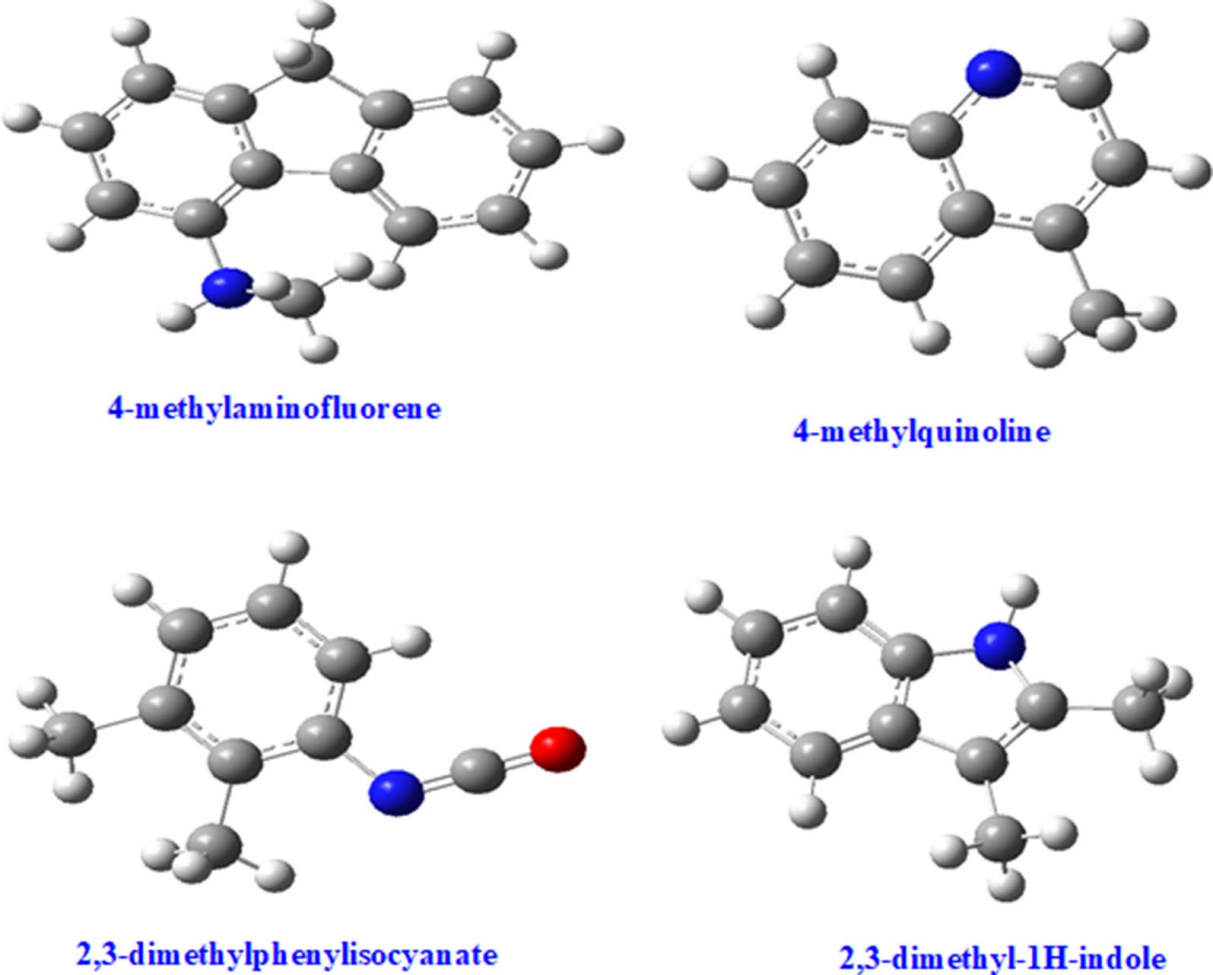
Reaction products from the oxidative pyrolysis of equimassic mixture of cellulose and tyrosine–the blue atoms represent nitrogen, red atom represents oxygen, carbon atoms are grey while white atoms are hydrogens

These compounds are reported for the first time in this study and are of interest to the combustion community, especially in the search for renewable energy resources from biomass materials. Similarly, nitrogenated chemicals in the class of both dioxin-like (4-methylquinoline) and dibenzofuran-like (2,3-dimethyl-1H-indole) chemicals are reported in Fig. 3a with the exception of 4-methylaminofluorene and 2,3-dimethylphenylisocyanate. The later chemical, 2,3-dimethylphenylisocyanate, falls into the isocyanate family of suspected carcinogens as well as mutagens (El-Zaemey et al. [38]). The nitrogenated analogues of dioxins and dibenzofurans reported in Figs. 3, 4 and  5 are emerging pollutants [37] as well as potential precursors for NOx during combustion if present in bio-oil or biodiesel. Therefore, this is a classic example of the possible negative economic and health impacts renewable energy can cause if large amounts of N-biomass is present in biomass model components targeted for bio-oil production.

From Fig. 3 (a), it is important to note that the evolution of most chemicals reaches a maximum between 300 and 400 °C for pyrolysis and between 250 and 350 °C for oxidative pyrolysis (cf. Fig. 3b). This is in agreement with various pyrolysis studies reported in literature on the thermal degradation of biomass materials. At temperatures in excess of 500 °C, the main product is biochar. It is interesting to observe that few compounds formed have a significant relationship with cellulose. This implies that tyrosine inhibits [6] the formation of chemicals that are of significant importance in the formation of bio-oil–levoglucosan, furfural, furan, and 5-hyroxymethylfurfural [14]. This finding suggests that in the production of bio-oil by the process of thermal degradation of cellulosic materials, any N-biomass material should be highly reduced or completely eliminated. Biomass materials containing high levels of amino acids are largely not good candidates for bio-oil.

The chemicals reported in Figs. 4 and 5 and Additional file 1: Figures S3 and S4, are unique because they are not formed when tyrosine is pyrolyzed individually [18] or when cellulose is pyrolyzed alone under similar conditions as applied in this work. This can only explain two scientific proposals; i.e., firstly, tyrosine or any model amino acid inhibits the formation of chemicals of interest for bio-oil production, and secondly, cellulose in presence of amino acids acts as a catalyst for the formation of nitrogenated chemicals, which are suggested as intermediates for drug and polymer manufacture [39, 40], in addition to the fact that they are emerging environmental pollutants having similar toxicological characteristics as polychlorinated dioxins and polychlorinated dibenzofuran analogues [37]. Therefore, this study has made some ground breaking informative findings towards the synthesis of intermediates of medical value and also offers direction against the inclusion of amino acids in lignocellulosic components during the production of bio-oil. Isocyanates, which are used as precursors for foam production, have been associated with serious health problems such as asthma and cancer (El-Zaemey et al. [38]) and, therefore, should be treated as unwanted products during the thermal degradation of a binary mixture of tyrosine and cellulose. Molecular compounds presented in Figs. 4 and 5 were modelled using Gaussian’09 computational platform.

We have also reported other high molecular reaction products from the binary pyrolysis of cellulose and tyrosine in our previous publication [6]. Despite concerted efforts by Ren and his co-workers [7], Ren et al., [7], there has been little research on the interaction of cellulose materials with model amino acid compounds that can lead to the formation of larger N-molecular compounds such as quinoline and N-aromatic compounds of environmental importance reported in this study. Nonetheless, (Kirkok et al. [6]) experimented on the co-pyrolysis of cellulose and tyrosine amino acid and obtained indole derivatives and 1-naphthylisocyanate.

Ultimately, the balance between technology and environmental degradation is one of the greatest challenges of the 21st century. The explosion in industrial activities coupled with environmental mismanagement has resulted in an exponential rise in environmental pollutants that have had grave health concerns to the general public. The large volumes of gaseous matter; CO_2_, CO, and NOx, and particulate matter emitted into the atmosphere in the wake of industrial events has not only caused global warming and poor visibility in major towns and cities but also precipitated drought, floods, the disappearance of ecological systems, and thriving of legacy respiratory diseases–chronic obstructive pulmonary disease (COPD), lung cancer, emphysema, asthma, and cancer of the throat. Consequently, unless there is a judicious balance between technology and/or industrialization and environmental protection, an ecological disaster awaits humanity.

## Conclusions

This paper has demonstrated that dioxin and dibenzofuran like compounds are formed during the pyrolysis of model amino acid (tyrosine) and cellulose. The thermal degradation of cellulose is known to produce chemicals of economic value; levoglucosan and formaldehyde, but its combustion in the presence of model N-compounds such as amino acids inhibit the formation of these compounds. Remarkably, the pyrolysis of a binary mixture of tyrosine and cellulose yields high levels of nitrogenated compounds, which can be used as precursors for the manufacture of medicinal drugs and polymer materials, however, also branded as harmful emerging pollutants because of their toxic characteristics, similar to those of polychlorinated dioxins and dibenzofurans (PCDD/Fs). Therefore, the presence of N-components (amino acids) in biomass materials impedes the production of clean biomass based-fuels and, subsequently, compromises clean energy combustion. Overall, a judicious balance between the production of bio-oil and side products resulting from amino acids present in plant matter should be taken into account to minimize economic losses and mitigate against negative public health impacts. Future research should emphasize on the mechanistic formation of dioxin and dibenzofuran-like heterocycles from the pyrolysis of a mixture of cellulosic materials and amino acids.

## Supplementary information

**Additional file 1: Figure S1.** Reactor assembly used in the thermal degradation of biomass composites (tyrosine and cellulose). **Figure S2.** Modelled structure of levoglucosan (right), and its corresponding 2-D structure (left). In the modelled structure, carbon atoms are grey, oxygen atoms are red while hydrogen atoms are white. **Figure S3.** Nitrogenated molecular products from the co-pyrolysis of cellulose and tyrosine - carbazole and 9H-pyrido[3,4-*b*]indole are dibenzofuran-like analogues considered emerging pollutants. **Figure S4.** Molecular products from the co-oxidative pyrolysis of cellulose and tyrosine - the chemicals are a mixture of nitrogenated and oxygenated heterocycles.

## Data Availability

Data will be made available upon request.

## Abbreviations

amu: Atomic mass unit
PCDD/Fs: Polychlorinated dioxins and dibenzofurans
COPD: Chronic obstructive pulmonary disease
TIC: Total ion current
MSD: Mass selective detector
STDS: System for thermal diagnostic studies
DFT: Density functional theory
EPR: Electron paramagnetic resonance
BDE: Bond dissociation energy
