# Supplementary material for: Dioxin and dibenzofuran like molecular analogues from the pyrolysis of biomass materials—the emerging challenge in bio-oil production
Source: BMC Chem. 2021 Jan 15;15(1):3. doi: 10.1186/s13065-020-00732-z (PMC7809783; doi:10.1186/s13065-020-00732-z)
Supplement: Supplementary file 1 — Additional file 1: Figure S1. Reactor assembly used in the thermal degradation of biomass composites (tyrosine and cellulose). Figure S2. Modelled structure of levoglucosan (right), and its corresponding 2-D structure (left). In the modelled structure, carbon atoms are grey, oxygen atoms are red while hydrogen atoms are white. Figure S3. Nitrogenated molecular products from the co-pyrolysis of cellulose and tyrosine - carbazole and 9H-pyrido[3,4-b]indole are dibenzofuran-like analogues considered emerging pollutants. Figure S4. Molecular products from the co-oxidative pyrolysis of cellulose and tyrosine - the chemicals are a mixture of nitrogenated and oxygenated heterocycles. [file 13065_2020_732_MOESM1_ESM.docx]

**Dioxin and dibenzofuran like molecular analogues from the pyrolysis of cellulosic biomass materials – The emerging challenge in bio-oil production**

Samuel K. Kirkok^1^, Joshua K. Kibet*^1^, Thomas Kinyanjui^1^, Francis I. Okanga^1^, Vincent O. Nyamori^2^

^1^Department of Chemistry, Egerton University, P.O Box 536-20115, Egerton

^2^School of Chemistry and Physics, University of KwaZulu-Natal, Westville Campus,

Private Bag X54001, Durban, 4000, South Africa

Correspondence: [jkibet@egerton.ac.ke](mailto:jkibet@egerton.ac.ke)

**Supporting information**

Herein, we supply information that would be useful in enhancing the understanding of the chemicals that are evolved during the thermal degradation of the binary mixture of cellulose and tyrosine under conditions that simulate thermochemical conversion. The reactor used in the degradation process is also presented in this section.


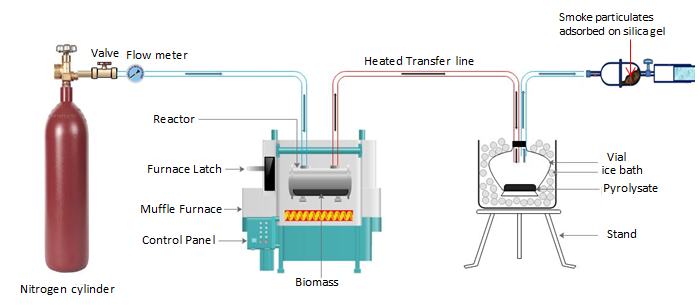


**S1** Reactor assembly used in the thermal degradation of biomass composites (tyrosine and cellulose)




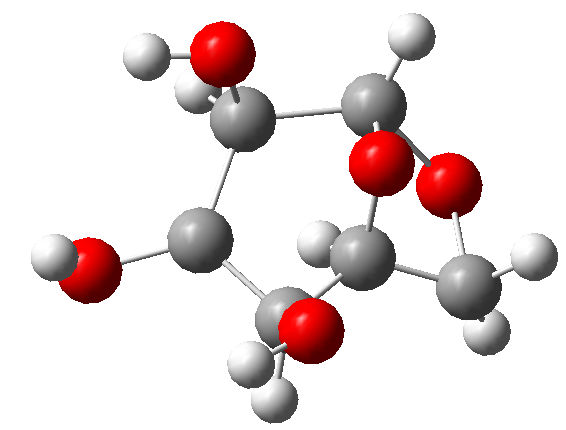





**S2** Modelled structure of levoglucosan (right), and its corresponding 2-D structure (left). In the modelled structure, carbon atoms are grey, oxygen atoms are red while hydrogen atoms are white.





**S3** Nitrogenated molecular products from the co-pyrolysis of cellulose and tyrosine - carbazole and 9H-pyrido[3,4-*b*]indole are dibenzofuran-like analogues considered emerging pollutants





**S4** Molecular products from the co-oxidative pyrolysis of cellulose and tyrosine - the chemicals are a mixture of nitrogenated and oxygenated heterocycles
